# Supplementary material for: Adverse Childhood Experiences Predict Common Neurodevelopmental and Behavioral Health Conditions among U.S. Children
Source: Children (Basel). 2021 Aug 31;8(9):761. doi: 10.3390/children8090761 (PMC8471662; doi:10.3390/children8090761)
Supplement: Supplementary file 1 [file children-08-00761-s001.zip › children-1352581-supplementary.pdf]

Supplemental

Table S1. Questions from the National Survey of Child Health used in analyses.

Adverse Childhood Experiences (ACE) Questions
To the best of your knowledge, has this child EVER experienced any of the following?
Parent or guardian divorced or separated
1 = Yes
2 = No

To the best of your knowledge, has this child EVER experienced any of the following?
Parent or guardian died
1 = Yes
2 = No

To the best of your knowledge, has this child EVER experienced any of the following?
Parent or guardian served time in jail
1 = Yes
2 = No

To the best of your knowledge, has this child EVER experienced any of the following?
Saw or heard parents or adults slap, hit, kick, punch one another in the home
1 = Yes
2 = No

To the best of your knowledge, has this child EVER experienced any of the following?
Lived with anyone who was mentally ill, suicidal, or severely depressed
1 = Yes
2 = No

To the best of your knowledge, has this child EVER experienced any of the following?
Lived with anyone who had a problem with alcohol or drugs
1 = Yes
2 = No

Since this child was born, how often has it been very hard to cover the basics, like food or housing, on your family's income?
1 = Never
2 = Rarely
3 = Somewhat often
4 = Very often

Health Condition/Diagnosis-Related Questions
Has a doctor or other health care provider EVER told you that this child has…
Autism or Autism Spectrum Disorder (ASD)? Include diagnoses of Asperger's Disorder or Pervasive Developmental Disorder (PDD).
1 = Yes
2 = No

Has a doctor or other health care provider EVER told you that this child has Attention Deficit Disorder or Attention-Deficit/Hyperactivity Disorder, that is, ADD or ADHD?
1 = Yes
2 = No

Has a doctor or other health care provider EVER told you that this child has Epilepsy or seizure disorder?
1 = Yes
2 = No

Has a doctor or other health care provider EVER told you that this child has Cerebral Palsy?
1 = Yes
2 = No

Has a doctor or other health care provider EVER told you that this child has Down Syndrome?
1 = Yes
2 = No

Has a doctor, other health care provider, or educator EVER told you that this child has Intellectual Disability (formerly known as Mental Retardation)? Examples of educators are teachers and school nurses.

1 = Yes
2 = No

Has a doctor, other health care provider, or educator EVER told you that this child has Developmental Delay? Examples of educators are teachers and school nurses.
1 = Yes
2 = No

Has a doctor, other health care provider, or educator EVER told you that this child has Learning Disability? Examples of educators are teachers and school nurses.
1 = Yes
2 = No

Has a doctor or other health care provider EVER told you that this child has Anxiety Problems?
1 = Yes
2 = No

Has a doctor or other health care provider EVER told you that this child has Depression?
1 = Yes
2 = No

Has a doctor or other health care provider EVER told you that this child has Substance Use Disorder?
1 = Yes
2 = No

Has a doctor, other health care provider, or educator EVER told you that this child has Behavioral or Conduct Problems? Examples of educators are teachers and school nurses.
1 = Yes
2 = No

Has a doctor or other health care provider EVER told you that this child has Tourette Syndrome?
1 = Yes
2 = No

Has a doctor, other health care provider, or educator EVER told you that this child has Speech or other language disorder? Examples of educators are teachers and school nurses.
1 = Yes
2 = No

Has a doctor or other health care provider EVER told you that this child has Frequent or severe headaches, including migraine?
1 = Yes
2 = No

Table S2. Tests of orthogonal polynomial contrasts for ACE scores predicting neurodevelopmental and behavioral outcomes.

	Linear Contrast		Quadratic Contrast		Cubic Contrast	
**Model Coefficients**						
ACE = 0	-1.211		1.107		-0.555	
ACE = 1	-0.499		-0.694		1.507	
ACE = 2	0.214		-1.257		-1.172	
ACE ≥ 3 (mean 3.8)	1.496		0.844		0.220	
	**F-Value**	**P-Value**	**F-Value**	**P-Value**	**F-Value**	**P-Value**
**Outcomes**						
Down Syndrome	0.67	0.4121	0.05	0.8257	0.04	0.8396
Tourette Syndrome	4.57	0.0325	1.80	0.18	0.01	0.9169
Cerebral Palsy	3.10	0.0782	2.04	0.1537	0.04	0.8477
Epilepsy	4.65	0.0310	10.05	0.0015	2.89	0.0894
Speech Disorder	18.13	<0.0001*	57.70	<0.0001*	0.40	0.5248
Autism Spectrum Disorder	11.65	0.0006*	29.92	<0.0001*	0.08	0.7760
Intellectual Disability	4.01	0.0453	12.49	0.0004*	9.09	0.0026
Headaches/Migraine	22.23	<0.0001*	126.68	<0.0001*	2.68	0.1014
Developmental Delay	50.09	<0.0001*	153.69	<0.0001*	16.13	<0.0001*
Learning Disability	46.16	<0.0001*	186.31	<0.0001*	9.06	0.0026
Attention-Deficit/Hyperactivity Disorder	79.41	<0.0001*	349.38	<0.0001*	19.41	<0.0001*
Anxiety Problems	91.78	<0.0001*	401.27	<0.0001*	39.61	<0.0001*
Behavior/Conduct Problems	120.52	<0.0001*	562.23	<0.0001*	49.03	<0.0001*
Depression	104.32	<0.0001*	473.99	<0.0001*	49.39	<0.0001*
Substance Use Disorder	3.49	0.0617	16.01	<0.0001*	12.19	0.0005*

* P value <0.001 (Bonferroni-corrected P < 0.05).
